# Supplementary figures and images for: Distinct adaptation and epidemiological success of different genotypes within Salmonella enterica serovar Dublin
Source: eLife. 2025 Jun 25;13:RP102253. doi: 10.7554/eLife.102253 (PMC12194135; doi:10.7554/eLife.102253)

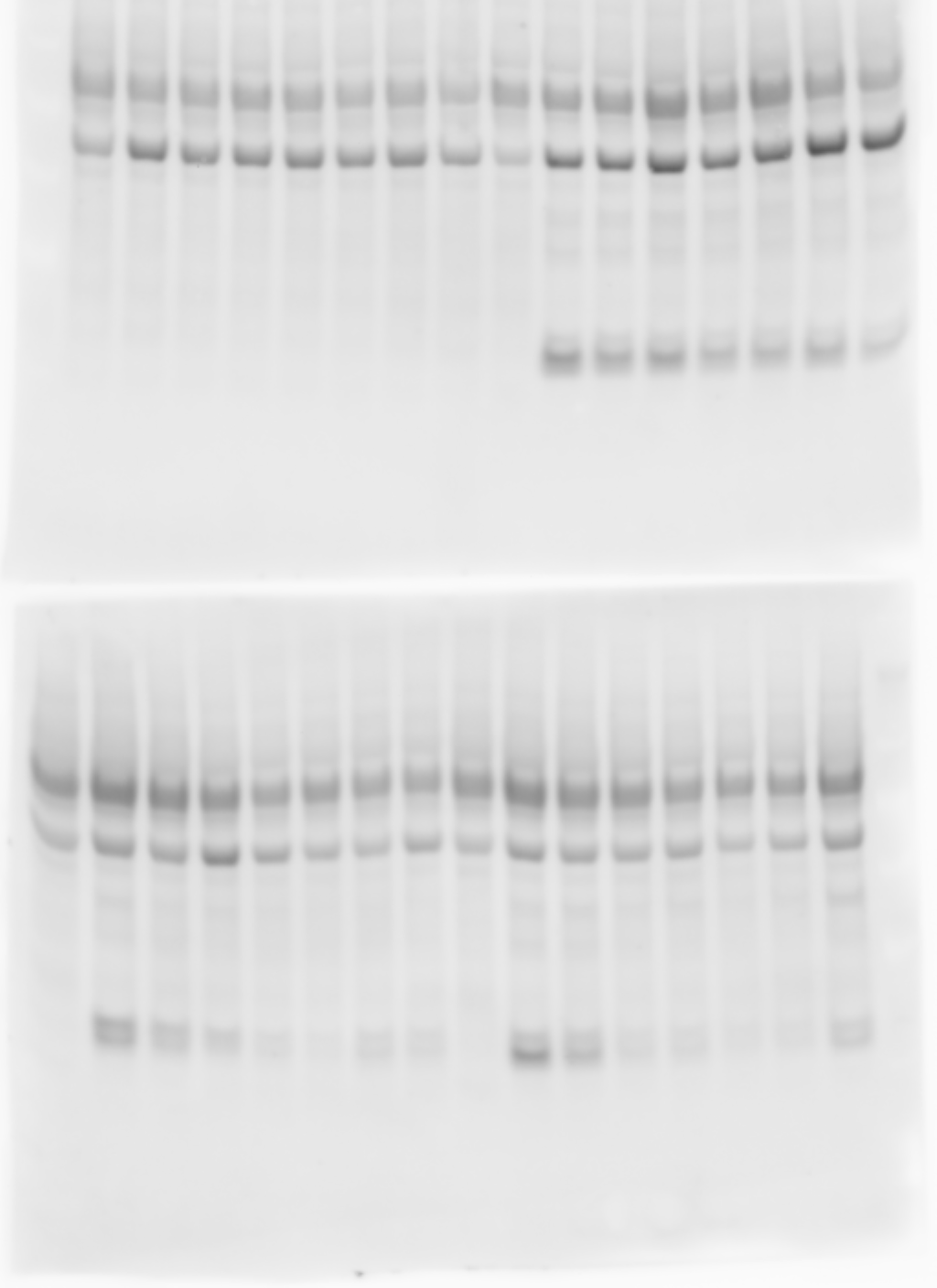

Supplement: Figure 6—source data 1. [file elife-102253-fig6-data1.tif]
