# Supplementary material for: Distinct adaptation and epidemiological success of different genotypes within Salmonella enterica serovar Dublin
Source: eLife. 2025 Jun 25;13:RP102253. doi: 10.7554/eLife.102253 (PMC12194135; doi:10.7554/eLife.102253)
Supplement: Figure 6—source data 2. [file elife-102253-fig6-data2.pptx]

## Slide 1
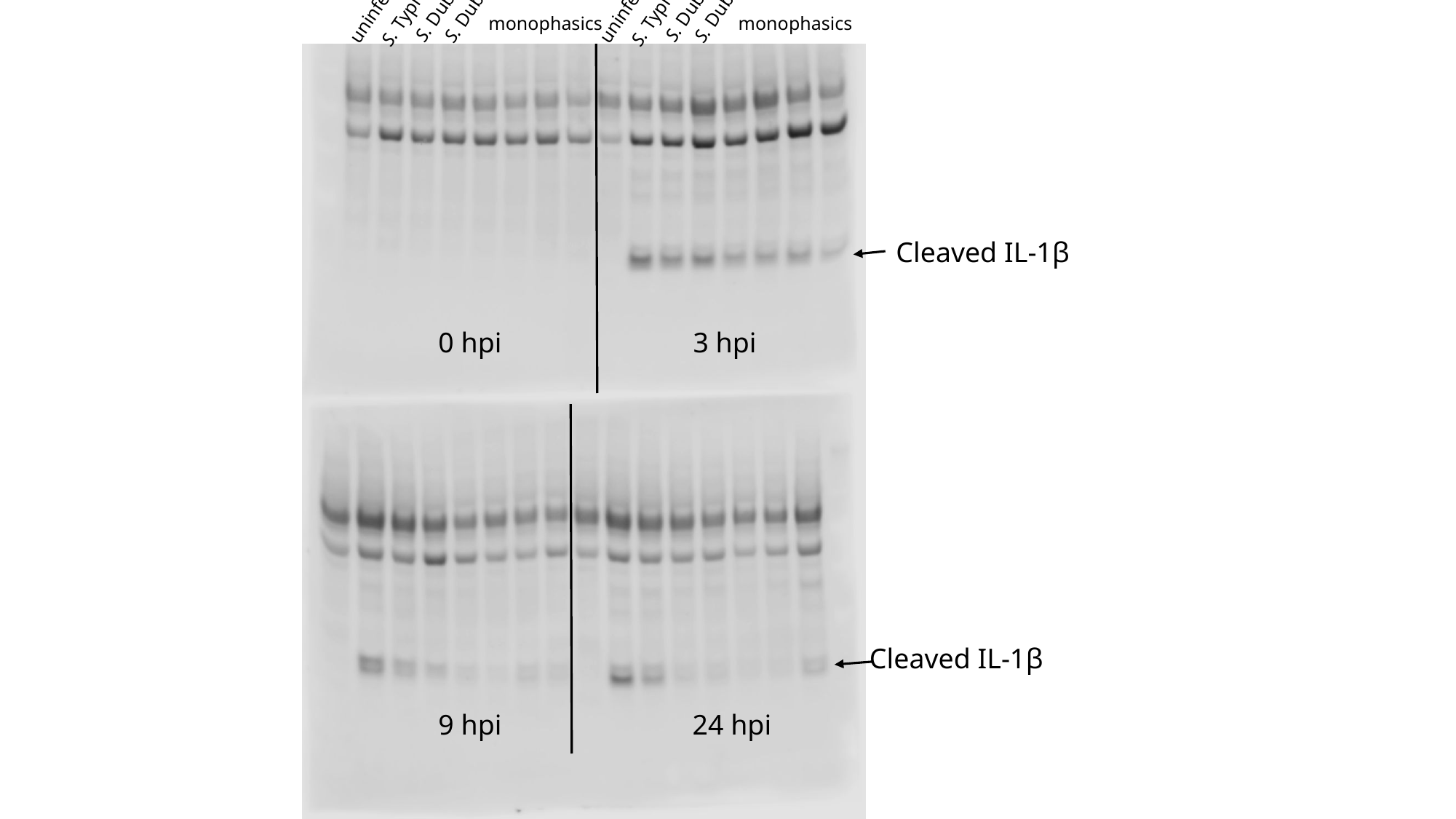

S. Typhimurium
S. Typhimurium
S. Dublin ST10
S. Dublin ST10
S. Dublin ST74
S. Dublin ST74
uninfected
uninfected
monophasics
monophasics
Cleaved IL-1β
0 hpi
3 hpi
Cleaved IL-1β
9 hpi
24 hpi
